# Unusual hypertrophic cardiomyopathy: case report of an early onset wild-type ATTR amyloidosis accompanied by a chromosomal duplication involving the *MYH6* and *MYH7* gene

**DOI:** 10.3389/fcvm.2025.1462523

**Published:** 2025-02-03

**Authors:** Jassin Hamidi, Yvonne Hanel, Sven Dittmann, Wanda Maria Gerding, Huu Phuc Nguyen, Karin Klingel, Eric Schulze-Bahr

**Affiliations:** ^1^ Institute for Genetics of Heart Diseases, University Hospital Münster, Münster, Germany; ^2^ Department of Human Genetics, Ruhr-University Bochum, Bochum, Germany; ^3^ Institute for Pathology and Neuropathology, University Hospital Tübingen, Tübingen, Germany

**Keywords:** hypertrophic cardiomyopathy, wtATTR, amyloidosis, copy number variation, *MYH6*, *MYH7*

## Abstract

**Background:**

Hypertrophic cardiomyopathy (HCM) is characterized by an increased left ventricular (LV) wall thickness and LV mass. With an estimated prevalence of 1:200–500, HCM is among the most common genetically determined cardiac diseases. Functionally, enhanced tissue stiffness and reduced elasticity, combined with diastolic dysfunction and myocardial fibrosis, can eventually lead to life-threatening arrhythmias and impaired blood flow through the heart chamber. Typical symptoms associated with HCM include atrial fibrillation (AF), syncope, ventricular fibrillation, and cardiac arrest. At the molecular level, various genetic and/or non-genetic etiologies can lead to HCM.

**Case summary:**

In this case, we report on a 60-year-old male patient with severe, progressive hypertrophic cardiomyopathy (HCM) in an uncommon and ambivalent setting. Right ventricular (RV) biopsy and multi-phase skeletal scintigraphy diagnosed transthyretin amyloidosis with cardiac involvement. Sanger sequencing of the transthyretin gene revealed a wild-type sequence. Phenotypically, the patient initially presented with syncopal episodes, atrioventricular (AV) block, and atrial fibrillation. Subsequently, bilateral carpal tunnel syndrome and polyneuropathy developed. However, the progressive and early onset of left ventricular hypertrophy did not align with the typical presentation of HCM in the context of ATTR. Therefore, next-generation sequencing (NGS) analysis revealed a rare chromosomal duplication in both cardiac myosin genes, *MYH6* and *MYH7*. Consequently, two distinct and rare disease entities co-occurred in this patient, both ultimately leading to HCM.

**Discussion:**

To date, no other case featuring wild-type transthyretin amyloidosis (wtATTR) concurrently with a chromosomal duplication affecting both cardiac myosin heavy chain genes has been reported in the literature. This highlights the extreme rarity of this condition, making it challenging to ascertain the extent to which a presumably mutated hybrid myosin gene construct or the TTR amyloid fibrils contribute to stiffness, tissue fibrosis, and cardiac dysfunction. Ultimately, both effects converged in this case, leading to the same cardiac disease with an exacerbated phenotypical outcome of hypertrophic cardiomyopathy (HCM). While early onset wtATTR is an uncommon clinical finding, another significant clinical condition was identified in this patient, marked by an unusual copy number variation (CNV) in the genes *MYH6* and *MYH7*.

## Introduction

Hypertrophic cardiomyopathy (HCM) is one of the most frequent and genetically determined cardiac disease with an estimated prevalence of 1:200–500 (1). HCM is characterized by an increased left ventricular (LV) wall thickness, with or without blood flow impairment due to obstruction, and increased LV mass. The thickened LV wall enhances tissue stiffness, reduces elasticity, and disrupts blood flow through the heart chamber. Notably, HCM predominantly occurs asymmetrically, with the greatest involvement typically seen in the basal interventricular septum below the aortic valve (2). Among the most frequent phenotypic symptoms are severe cardiac arrhythmia, heart failure and/or sudden cardiac death, which depends on the severity and progression of the disease. Histologically, HCM is primarily characterized by the development of myocellular disarray and myocardial fibrosis, which lead to an adverse tissue reorganization (3). Genetically, HCM is primarily inherited in an autosomal dominant pattern. To date, 40 different genes have been evaluated for disease-gene relationships in ClinGen, with 16 of these genes demonstrating definitive strong to moderate disease gene validity. However, the penetrance and severity of HCM symptoms vary significantly among different genetic subtypes and even within the same family, despite sharing a specific HCM gene mutation. This variability may be influenced by the co-occurrence and interaction of other genetic and non-genetic factors, such as age (2). HCM genes encoding sarcomere-associated proteins account for the majority of all genetically resolved HCM cases whereas around 40% of all HCM cases remain unresolved (2). Mutant sarcomere proteins *β*-myosin heavy chain (*MYH7*) and myosin-binding protein C (*MYBPC3*) represent the most prevalent genetic subtypes, accounting for approximately 50% of familial HCM cases (4). Othersubtypes may include mutations in other sarcomere-associated genes and proteins, such as *TNNT2*, *TNNI3*, *TPM1*, or *ACTC1*, or syndromic disorders with HCM, including lysosomal or glycogen storage disorders. On the other hand, α-myosin heavy chain (*MYH6*) variants have also been reported in some cases of HCM in recent years (5).

Transthyretin (TTR) is a small, 147 amino acid-protein primarily produced by the liver and plays a crucial role in the blood transport and tissue distribution of thyroid hormones and holoretinol binding protein (RBP). Acting as a carrier protein, the native TTR forms homotetramers under normal conditions and is composed of four beta sheets. Mechanistically, TTR binds to thyroxine and RBP to protect them from degradation for efficient delivery into the target organs and tissues. However, mutations in the encoding *TTR* gene on chromosome 18q12.1 can lead to misfolding and cellular aggregation of the TTR protein, resulting in various clinical tissue manifestations of amyloidosis, mainly as cardiomyopathy form (HCM), kidney insufficiency and neuropathy. However, inherited forms of ATTR (mATTR) are rare (estimated: 4:100,000) (6). In contrast, wild-type transthyretin amyloidosis (wtATTR) is more common and is characterized by the absence of a mutation (pathogenic variant) in the TTR gene, the only associated gene to date. wtATTR has a similar clinical manifestation to mutant ATTR (mATTR). Transthyretin-related forms of amyloidosis (ATTR), however, are uncommon as well as serum amyloid A-related amyloidosis (AA amyloidosis), seen during chronic inflammatory disorders such as rheumatoid arthritis, Crohn's disease, or colitis ulcerosa. The most common form (nearly 70%) of amyloidosis is the so-called AL amyloidosis (light-chain associated amyloidosis). In TTR-related amyloidosis, the mechanisms underlying the kinetic instability and monomerization of the native TTR protein, leading to denaturation and misassembly with deposits of amyloid fibrils, are only partially understood (7). Similarly to mATTR, native TTR proteins can lead to the cardiac infiltration and deposits of TTR amyloid fibrils and subsequently to stiffness, tissue fibrosis and organ dysfunction in the setting of cardiac hypertrophy. Typically, wtATTR-CM has a late onset and is almost exclusively found in patients with an age of onset >70 years, showing an often under recognized form of heart failure. Males are typically more frequently affected by wild-type transthyretin cardiomyopathy (wtATTR-CM) than females. Other clinical features include a higher incidence of bilateral carpal tunnel syndrome, spinal stenosis, and spontaneous biceps tendon rupture (8–11). Epidemiologically, the incidence of ATTR-CM is estimated to be 1: 10,000 in the elderly population above 65 years of age (12).

## Case description

A 41 years-old male patient (Table 1) suffered from dizziness and a short syncopal attack during a car ride. After hospital admission, an atrioventricular block of third degree (AVB III°) was diagnosed and subsequently, a dual-chamber and frequency-adaptive pacemaker was implanted. Four years later, in 2004 and in January of 2005, a paroxysmal episode of atrial fibrillation (AF) was first detected by device interrogation of the dual-chamber aggregate and terminated by propafenone. Subsequently, a beta-blocker therapy with bisoprolol (5 mg/day) was initiated. In transthoracic echocardiography, a septal left ventricular hypertrophy was first identified.

**Table 1:** Basic patient information.

Basic information	
Sex	Male
Age	60
Previous medical history (before 2001)	None
Alcohol intake	Occasionally
Asthma	None
Blood pressure (systolic/diastolic)	140/90 mmHg
Arterial hypertension	Yes
Body mass index (BMI)	30.5 kg/m²
Chronic kidney disease	None
COPD	None
Coronary heart disease	None
Albumin	4.7 g/dl
Creatinin	1.02 mg/dl
Hemoglobin	15.7 g/dl
Diabetes	None
Dialysis	None
Drug abuse	None
Family history of stroke	None
Family history of heart failure, arrhythmias, cardiomyopathy, sudden cardiac death	Brother (unknown SCD at 18 years). Mother suffered from heart problems
Gastrointestinal bleeding history	None
Gout	None
History of cancer	Squamous cell carcinoma of the skin, surgically removed. No evidence of metastases
HIV status	Negative
Liver cirrhosis or steatosis	None
Smoker	Smoking occasionally until 2000
Stroke	No

To prevent further arrhythmic episodes the beta-blocker therapy was adjusted to 10 mg/day. For the next six years, the patient had no severe complaints or arrhythmias (Table 2).

**Table 2:** Timeline.

Timeline	
Year 2001	Dizziness and syncopal attack during car drive. Diagnosed with atrioventricular block of third degree (AVB III°). Implantation of a frequency-adaptive (DDDR) pacemaker aggregate
March 2004	Dizziness and syncopal attack. Detection of atrial fibrillation (AF) by the DDDR aggregate. Intake of propafenone to treat AF
January 2005	Detection of AF by the DDDR aggregate and intake of propafenone to treat AF. Stopping of AF after 48 h. Starting of treatment with bisoprolol (5 mg/day)
March 2005	Suspected non-obstructive hypertrophic cardiomyopathy (HCM) by echocardiography evaluation. Adjustment of beta-blocker (bisoprolol) therapy to 10 mg/day
December 2011	Recurrence of AF. Confirmation of pathologic left ventricular (LV) thickening up to 19 mm by echocardiography. Still normal diastolic function of LV. Medication: bisoprolol 10 mg/day, simvastatin 10 mg/day, magnesium, disruption of aspirin 100 mg/day treatment und replacement with phenprocoumon for 6 weeks
November 2019	Distal symmetrical small-fiber polyneuropathy in both feet. Initiation of pregabalin treatment (100 mg/day), additionally treatment with novaminsulfon in acute periods of pain. Further, bilateral carpal tunnel syndrome
January 2020	Gated-SPECT myocardial scintigraphy with regular nuclide accumulation in the LV and during stress protocol without evidence of a restricted perfusion reserve. No evidence of myocardial fibrosis. Absolute arrhythmia with atrial fibrillation and pacemaker interaction on exercise ECG
February 2020	Transesophageal echocardiography with a slightly enlarged left atrium (51 mm) and massive septal left ventricular hypertrophy (partially 37 mm septal wall thickness)
April 2020	Adjustment of medication: bisoprolol 12.5 mg/day, digimerck 0.07 mg/day, allopurinol 300 mg/day, edoxaban 60 mg/day
November 2020	Electroneurographic evidence of bilateral sensorimotor carpal tunnel syndrome associated with bilateral demyelinating neuropathy of the tibial nerve
December 2020	Suspected transthyretin amyloidosis. Sequencing analysis of TTR gene resulted in wild-type TTR sequence
February 2021	Multiphase skeletal scintigraphy from thorax region under application of 540 MBq ^99m^Tc + HDP indicating cardiac amyloidosis (Perugini score: >2)
March 2021	Detection of increased free κ-light chain (21.7 mg/L) and increased kappa/lambda ratio of 4 in patient's blood, indicating a monoclonal gammopathy and thereby a potential AL ATTR
April 2021	Multi-gene-panel sequencing revealed a pathogenic heterozygous copy number variation (CNV) with large duplication of exon 1–32 of myosin heavy chain alpha (*MYH6*) until exon 34–40 of myosin heavy chain beta (*MYH7*) genes. Optical genome mapping (OGM) revealed the size of the CNV of approximately 29,877 kb. Confirmatory Sanger sequencing and qPCR
May 2021	Heart insufficiency NYHA II, NT-proBNP: 878 ng/L. Echocardiographic evaluation exhibited strong hypertrophy of LV with slightly diminished ejection fraction with hypokinesis of mediobasal wall areas. Slightly mitral to medium trikuspid valve insufficiency with annulus dilatation. Positive echocardiographic parameters for heart involvement in amyloidosis
June 2021	Examination of endomyocardial biopsy from right ventricle. Histological detection of cardial amyloidosis with focal and diffuse amyloid pattern. Positive immunohistological staining for transthyretin. Negative staining for different lambda and kappa light chain antibodies. Altogether wtATTR-CM. Start of tafamidis treatment (61 mg/day)
March 2023	Follow up: decrease of NT-proBNP to 518 ng/L. Until now no further deterioration of the patient's conditions

**Figure 1:**
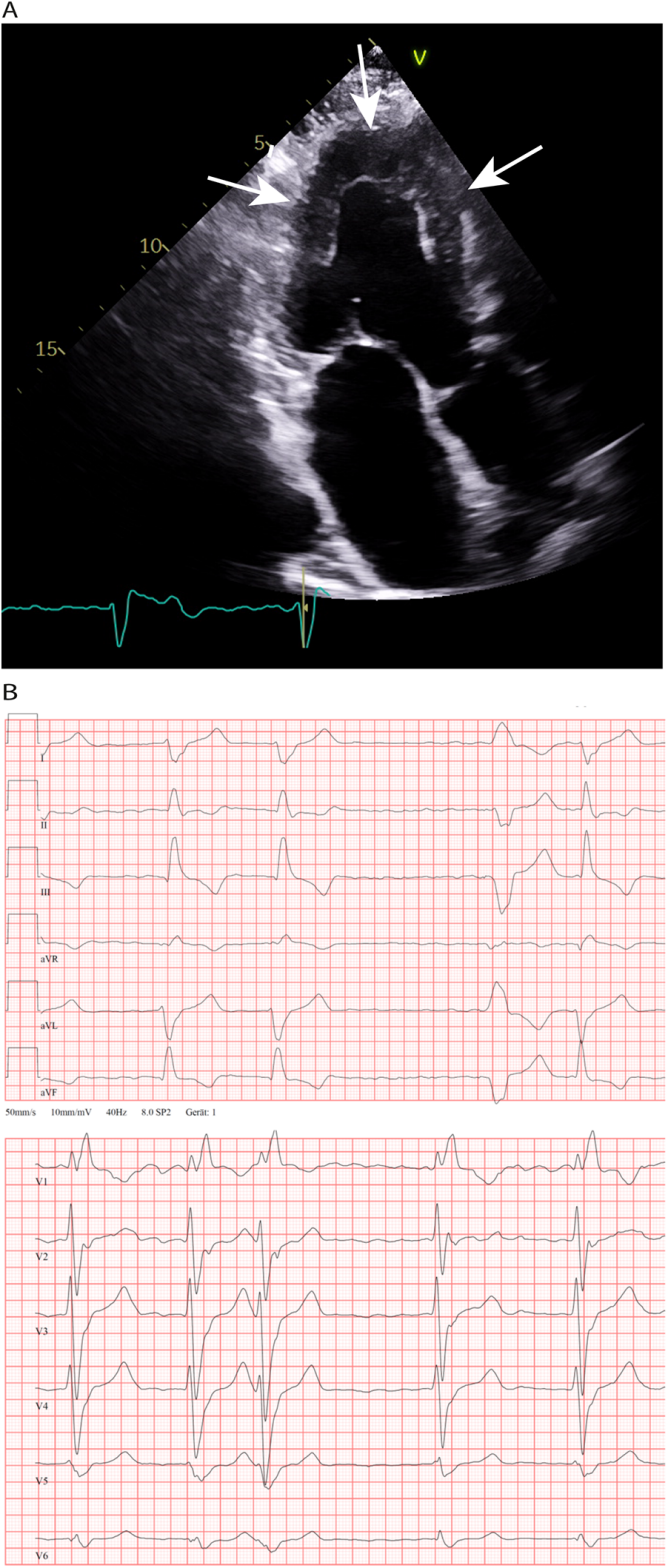
**(A)** Transthoracic echocardiography and 4-chamber view made in 2011; concentric LV hypertrophy (19 mm wall thickness) an LA enlargement. **(B)** Chest leads during an episode of atrial fibrillation at the age 60 years. Right bundle branch block, in addition marked right axis deviation (not shown) and absence of low voltage QRS complexes.

In December 2011 at the age 45-years, the patient began to suffer from persistent AF (CHA_2_DS_2_VASc score: 1). After echocardiographic examination, a pathologic LV wall thickening up to 19 mm was detected supporting the suspected diagnosis of HCM (Figure 1A). Nevertheless, the diastolic function of the LV was still normal. In the absence of arterial hypertension and with an apparent patchy myocardial tissue structure, Fabry disease was initially suspected; however, GLA activity was found to be normal. Following the exclusion of intracardiac thrombi, an external electric cardioversion was performed. Additionally, coronary angiography ruled out the presence of coronary artery disease. Anticoagulant treatment with phenprocoumon (INR values between 2.0 and 3.0) was initiated; for the next 8 years, the patient had no cardiac complaints (Table 2).

In November 2019, at age 53-years, the patient was diagnosed with a distal, symmetrical small-fiber polyneuropathy in both legs. Subsequently, a medication of pregabalin (100 mg/day) was initiated and, during acute periods of pain, novaminsulfon treatment was accompanied. Of note, a carpal tunnel syndrome was also noted in both arms. Due to symptoms of angina pectoris, gated-SPECT myocardial scintigraphy was performed, which revealed no evidence of ischemia or perfusion deficits at rest or during pharmacological stress, and no indication of myocardial fibrosis.

During an exercise ECG in January of 2020, however, the patient suffered from bradycardic atrial fibrillation and pacemaker interaction. Transesophageal echocardiography was conducted showing an enlarged left atrium (51 mm). More interestingly, the initial assumption of a HCM was now confirmed by detection of a progressive and massive septal LV hypertrophy (partially 37 mm wall thickness). LV ejection fraction was diminished and approximately 50%. Additionally, a spherical enlarged structure was detected in the area of the apical ventricular septum. In an exercise electrocardiogram, the patient reached 125 W until physical exhaustion without any dyspnoea; the heart rate increased from 63 to 101 bpm without any arrhythmic events. The daily medication was adjusted to 12.5 mg/day bisoprolol (1.5-0-0.5), 0.07 mg/day digitoxin (1-0-0), 300 mg/d allopurinol (1-0-0) and 60 mg/d edoxaban (1-0-0) to treat possible recurring symptoms of arrhythmia, embolism, heart insufficiency and emerging small-fiber polyneuropathy.

Eleven months later, in November 2020, electroneurographic evaluation revealed bilateral sensorimotor carpal tunnel syndrome in conjunction with bilateral demyelinating neuropathy of the tibial nerve. To determine the underlying cause of the emerging polyneuropathy, amyloid polyneuropathy due to transthyretin amyloidosis was suspected, necessitating sequencing of the transthyretin (TTR) gene. Sanger sequencing of the entire transthyretin gene (OMIM176300, NM_000371), including all intron splice sites, was performed. This analysis revealed a complete wild-type TTR gene sequence, indicating the absence of an inherited form of transthyretin amyloidosis.

Due to suspected amyloidosis, multiphase skeletal scintigraphy of the thoracic region was performed in February 2021, utilizing 540 MBq of ^99m^Tc + HDP. Initially, an enlargement of the heart was observed (5 min post-infusion). At 2 h post-infusion, a diffuse increase in radionuclide tracer activity in the myocardium was detected, corresponding to a Perugini Score of 2, indicating cardiac involvement by amyloidosis. Additionally, evidence of active polyarthrosis was noted. One month later, a blood sample from the patient showed an increase in free kappa light chains (21.7 mg/L) and an elevated kappa/lambda ratio of four. This finding was suggestive for an AL amyloidosis. Additionally, there was also an elevation in NT-proBNP levels (878 ng/L). For histological confirmation of amyloidosis and to investigate a possible AL amyloidosis, biopsies were obtained during gastroscopy and coloscopy. However, the obtained tissue material did not reveal any amyloid deposits after staining. Meanwhile, echocardiographic evaluation confirmed a pronounced LV hypertrophy of LV with slightly diminished ejection fraction and mediobasal hypokinesis, also with dilation of the left atrium (LA), right atrium (RA) und right ventricle (RV) and absence of a pericardial effusion. Medical treatment was adjusted to 5 mg/day bisoprolol, (1-0-0), 60 mg/day edoxaban (1-0-0), 300 mg/day allopurinol (0-0-1) and magnesium supplementation (1-1-1).

**Figure 2:**
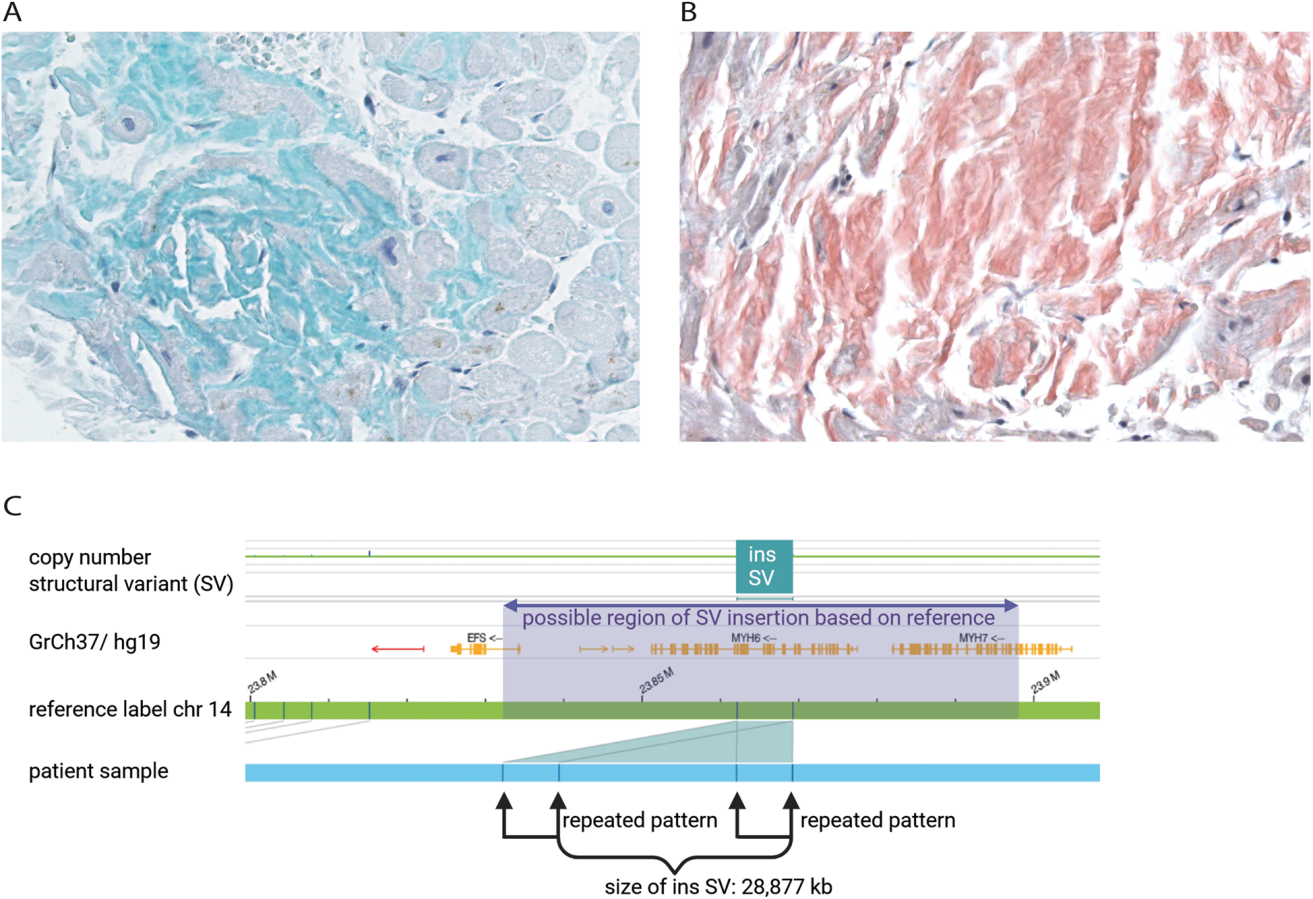
Immunohistochemical staining of transthyretin (green) of patient's endomyocardial biopsy from right ventricle representing the affected area of wtATTR **(A)** Kongo-red staining of patient's endomyocardial biopsy to determine amyloid structure of protein aggregates (red). Microscopic evaluation of the affected area revealed minor interstitial fibrosis and degenerative alterations of the myocardial tissue surrounded by fibrillary structures **(B)** optical genome mapping (OGM) exhibited an approximately 28,877 kb heterozygous insertion as structural variant on chromosome 14q11.2, which may partially affect the *MYH6* and/or the *MYH7* gene **(C).**

**Figure 3:**
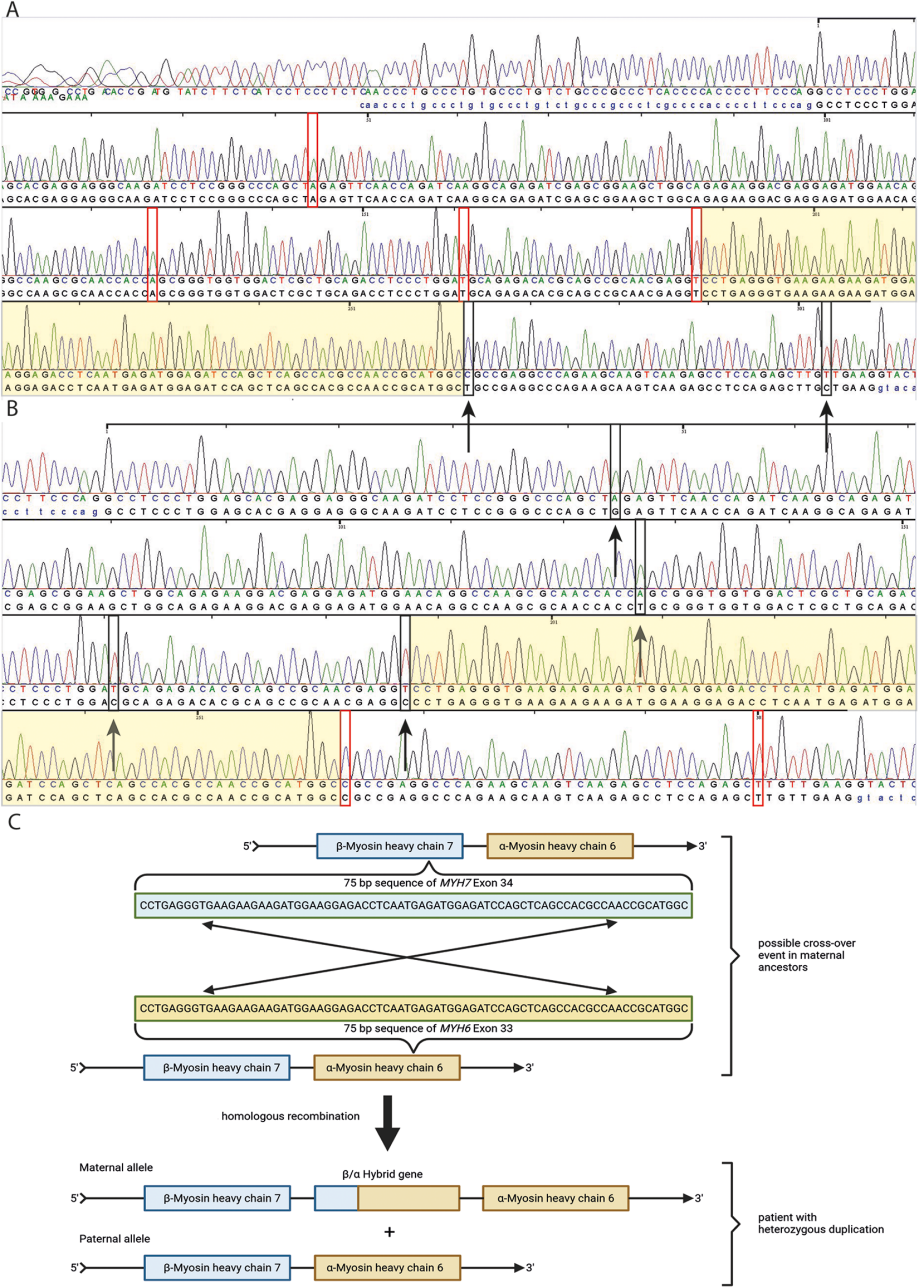
Sanger sequencing of *MYH6* exon 33 and *MYH7* exon 34 from patient's DNA (**A,B**). In **(A)**, red rectangles represent four base pairs of *MYH6* exon 33 which differ from those in *MYH7* exon 34. Black rectangles with black arrows represent two base pairs, which seem mutated and represent wild type sequence of *MYH7* exon 34 **(A).** In **(B)**, black rectangles with black arrows represent four base pairs, which seem mutated for *MYH7* exon 34 and differ from those in *MYH6* exon 33. Red rectangles represent two base pairs, which are wild type for *MYH7* exon 34 and differ from those in *MYH6* exon 33 **(B)** The light yellowish sequence represent an area of a possible cross-over event of *MYH6* exon 33 and *MYH7* exon 34 based on Sanger sequencing results of both exons and the position of base pair mismatches with the reference in these exons indicated by red and black rectangles. In **(C)**, a schematic illustration is shown of a possible cross-over event in a 75 bp long sequence area of *MYH6* exon 33 and *MYH7* exon 34 in maternal ancestors of the patient based on family history of heart failure and Sanger sequencing results of patient DNA **(C)**.

In April 2021, due to the early and progressive onset of wtATTR, a multi-gene panel sequencing approach using next-generation sequencing (NGS) technology was performed (Illumina TruSight Cardio panel, 174 genes). This revealed a rare structural variant after bioinformatic copy number variation (CNV) analysis. A heterozygous duplication of a gene region encompassing the alpha-myosin heavy chain 6 gene (*MYH6*) as well as the beta-myosin heavy chain 7 gene (*MYH7*) was detected. The duplication included exons 34–40 of *MYH7* and exons 1–32 of downstream *MYH6* gene. Both gene loci reside on immediate proximity on chromosome 14q11.2. For secondary CNV validation, a qPCR addressing the sequence transition between exon 31 and 32 of *MYH6* was performed and confirmed this structural variation. Exons 33 and 34 of *MYH6* were also analysed via qPCR, but were found in a diploid state (not duplicated). Using optical genome mapping (OGM), an approximately 29,877 kb heterozygous insertion on chromosome 14q11.2, which partially affects the *MYH6* and/or the *MYH7* gene, was identified as a larger, structural variant (Figure 2C). Since OGM is not a sequence reading method *per se*, the insertion cannot be precisely determined but it affects genomic chromosomal positions g.23862123 and g.23869233 on chromosome 14 that were found duplicated according to the labelling pattern. Due to low label density, SV calls in cases of small duplications are reported as insertions. Thus, a specific estimate about the exact intragenic position of the insertion cannot precisely be made, but the labelling pattern strongly hints to a duplication affecting both genes with the aforementioned exons. Remarkably, further investigation by Sanger sequencing of the affected chromosomal region revealed a most likely crossover event between exon 33 of *MYH6* and exon 34 of *MYH7* potentially resulting in a fusion hybrid gene of both myosin proteins (Figures 3A–C). In conclusion, a rare chromosomal duplication of both cardiac myosin genes, combined with the early-onset of a wtATTR, ultimately led to the diagnosis of HCM in accordance with the European Society of Cardiology (ESC) guidelines. Following the ACMG guidelines, this CNV was currently evaluated as variant of unknown significance (VUS) for HCM as ACMG class 3 (criteria: PM2_supporting, PS4_supporting, PP4_supporting).

In June of 2021, the patient underwent an endomyocardial right ventricular biopsy for evaluation of cardiac involvement by wtATTR. Post-interventional pericardial effusion was excluded. Microscopic evaluation of the biopsied tissue revealed minor interstitial fibrosis and degenerative alterations of the myocardial tissue accompanied by fibrillary structures with a positive Kongo-red staining (Figure 2B). Additionally, immunohistochemical staining of the affected area was positive for transthyretin (Figure 2A). The diagnosis of severe cardiac wild-type transthyretin amyloidosis was finally confirmed, characterized by both focal and diffuse amyloid deposition patterns. There was no histological evidence for a hemochromatosis, glycogenosis as well as an acute or chronic lymphocytic myocarditis. Remarkably, immunohistochemical staining for the κ- and λ-light chain antibodies was negative in the obtained myocardial tissue. In consequence, treatment with tafamidis (61 mg/day (1-0-0) was initiated due to presence of a wtATTR-CM. Furthermore, an ECG investigation revealed an episode of acute AF at the day of procedure (Figure 1B).

During follow-up evaluation in March 2023, a decrease of NT-proBNP (518 ng/L) were noted with a clinical improvement of the physical condition of the patient when compared with 2 years ago (Table 2). The daily current medication was now 5 mg/day bisoprolol (1-0-1), 25 mg/day ramipril (1-0-0), 300 mg/day Allopurinol (0-0-1), 60 mg/day edoxaban (1-0-0), 61 mg/day tafamidis (1-0-0) and 300 mg/day pregabalin (1-0-1). Given the positive family history for sudden cardiac death (brother, at the age of 18) and the recent echocardiographic findings, the patient was advised to replace the DDDR aggregate to a dual-chamber transvenous implantable cardioverter-defibrillator (ICD) system. However, this has not been conducted yet.

## Discussion

In the present case, we describe a very rare combination of two separate disease entities co-occurring in the same patient both causing left ventricular HCM. Initially, diffuse symptoms beginning with dizziness and an atrioventricular block of third degree (AVB III°) occurred followed by recurring episodes of persistent atrial fibrillation, together with a concentric LV hypertrophy suggestive for an ATTR-CM. However, a *TTR* gene mutation was not detected. A multi-gene-panel sequencing approach revealed a rare CNV in heterozygous state comprising a chromosomal duplication of parts of the *MYH6* gene as well as the adjacent *MYH7* gene. Both myosin heavy chain genes have already been known to cause or to be associated with HCM [*MYH7*: gene definitive causative for HCM (13, 14); *MYH6*: limited disease evidence (5, 15)]. Of note, a large duplication of this chromosomal area comprising both, *MYH6* and *MYH7*, has been reported to segregate in a familial case of HCM (16), but with a different duplication breakpoint (at exon 27 of both myosin genes). Although the described duplication may has the same molecular disease mechanism like the current case, it does not match the ACMG criteria for segregation or variant prevalence in phenotype-positive mutation carriers compared to controls due to different genomic breaking points within both genes. Particularly for the analysis of large duplications with uncertain functional consequences on the pathogenicity, the evaluation according to the ACMG criteria remains to be challenging, also due to a limited number of detected cases.

On the other hand, the histological and immunohistological evaluation of a RV endomyocardial biopsy was diagnostic for a cardiac ATTR-CM with a focal but also diffuse amyloid pattern. Recently, this condition was associated with severe forms of CM; especially demonstrating that amyloid CMs leading to poor survival rates among some patients. However, wtATTR-CMs leaded to better long-term prognosis compared to mATTR-CMs (17). In a cohort of 2,251 Caucasian patients with different forms of CMs, the sex-specific prevalence of CMs caused by wtATTR revealed a higher amount of male patients compared to light-chain amyloidosis CM patients (86.8% vs. 59.4%). Remarkably, the mean age of patients with wtATTR was 74.6 years highlighting the scarcity of wtATTR patients under the age of 60 years and below as described in this case (17). In line with this, first severe neuronal symptoms and diagnostic evidence indicating a transthyretin amyloidosis with cardiac involvement were already observed below 60 years of age in the current case. Considering the co-occurrence of both pathological conditions with the genetically determined CNV involving both myosin heavy chain genes and secondly the histologically and immunohistochemically detected wtATTR, the predominant driver of HCM cannot be determined. Presumably, both conditions mutually enhance the phenotypical progressive manifestation of HCM, particularly since each condition independently can cause severe forms of HCM or other hereditary heart defects (18–20). Most likely, the initial development of pathologic LV wall thickness is caused by the congenital genetic burden of the mutated *MYH6* and *MYH7* genes in this case, since the first cardiac symptoms have already occurred in year 2001 at the age of 40 years. Notably, the onset of neurological complaints, such as distal symmetrical small-fiber polyneuropathy in both feet and bilateral carpal tunnel syndrome, can be perceived as the initial manifestation of wtATTR-related symptoms which appeared almost 20 years later, in 2019 and shortly before. These neurological symptoms are common for ATTR amyloidosis with an overall prevalence of around 70%. Especially in early-onset patients, polyneuropathy is a predominant condition besides cardiac dysfunction (21). However, wtATTR patients who exhibit pronounced neurological symptoms as the initial indication of ATTR amyloidosis at the age of 58–60 years are very rare in the literature, as the typical age of onset for wtATTR symptoms is around 75–80 years. Remarkably, the commonly described symptoms in most wtATTR patients are heart disease and carpal tunnel syndrome, rather than sensorimotor polyneuropathy, as observed in this case (21, 22). The assumption that the mutated myosin genes primarily caused the initial cardiac symptoms, such as AF and recurrent syncope, is supported by the fact that typical wtATTR patients exhibit an initially indolent disease course, followed by rapid deterioration with severe cardiac dysfunction. Nevertheless, the progression of wtATTR is slower and has a higher median survival compared to light chain (AL) or ATTR amyloidosis (23). However, it has to be taken into consideration that the risk of cardiac symptoms with severe and live threatening arrhythmias will rise with increasing age and disease progression in the described patient.

Currently, no other case harbouring two distinct rare disease entities (wtATTR and mutant myosin heavy chain genes) has been described in the literature so far. This circumstance emphasizes the rarity of this medical condition in which it is hard to determine to which extent a presumably mutated hybrid myosin gene construct or the TTR amyloid fibrils are leading to stiffness, tissue fibrosis and organ dysfunction of the heart. Unfortunately, it was not possible to stain the biopsied tissue with *MYH6* and *MYH7* antibodies to reveal a possible co-localization of *MYH6* and *MYH7* fluorescence signal indicating a dysfunctional hybrid protein construct. Sonoda et al. described a similar case of an infant with atrial septal defect and atrial arrhythmias harbouring a large chromosomal deletion comprising also both cardiac myosin genes (*MYH6* and *MYH7*). In this case, it was speculated that the genetic consequence may leaded to a new hybrid protein consisting of *MYH6* and *MYH7* under the promotor of *MYH7* since the deletion was in frame. Unfortunately, a biopsy from this patient was not available to confirm this hypothesis (20). Singer et al. recently described another case of a large duplication affecting also *MYH6* and *MYH7* in a patient with HCM and no alternate genetic diagnosis. Although the exact breakpoints of this duplication could not be determined, the genetic mechanism leading to the duplication seemed to be similar to the current case. The authors described a remarkable 99.4% sequence identity between exon 26 of *MYH6* and exon 27 of *MYH7* which apparently caused a homologous recombination by misaligning the aforementioned exons (19). Notably, a similar high sequence identity between exon 33 of *MYH6* and exon 34 of *MYH7* was identified in the current case as well, proposing the same genetic consequence of a large duplication leading to a possible dysfunctional hybrid protein construct. Furthermore, according to the Sanger sequencing results we were able to localize the area of a presumable homologous recombination event in the described duplication in a small 75 bp segment of complete sequence identity between exon 33 of *MYH6* and exon 34 of *MYH7*. The fact that the described male patient also suffered from HCM might indicate that such chromosome 14-duplications involving both cardiac myosin genes, being presumably under the control of the MYH7 promotor, may contribute to more severe forms of HCM.

## Conclusion

In this case, a patient with severe, progressive HCM was characterized. Cardiac amyloidosis was diagnosed through right ventricular (RV) biopsy and multi-phase skeletal scintigraphy. The presence of increased free κ-light chain, indicative of a monoclonal gammopathy, implied an AL amyloidosis in the absence of multiple myeloma. Additionally, the development of AV block, atrial fibrillation, bilateral carpal tunnel syndrome, and polyneuropathy clearly indicated a wtATTR. Ultimately, Sanger sequencing analysis revealing wild-type transthyretin amyloidosis (wtATTR) without a TTR gene mutation. However, the progressive and early onset of left ventricular hypertrophy, along with the absence of a low voltage QRS complex, did not align with the typical features of HCM in the setting of ATTR. While early onset wtATTR is a rare diagnostic finding, another adverse clinical condition was considered in this case to explain the observed severe disease progression. Consequently, a large duplication in two cardiac myosin genes (*MYH6* and *MYH7*) has been identified upon extended genetic analysis which is an unusual, but aggravating finding with potential implications for further family cascade screening. On the other hand, with the severe early onset wtATTR, a second genetic hit should be considered—vice versa, this may raise the question whether a wtATTR-CM is still non-genetic. Here, both effects overlapped and it remains difficult to determine to which extent a presumably mutant hybrid myosin gene construct or the abnormal TTR amyloid fibrils led to organ dysfunction in this case. This circumstance emphasizes the rarity of this special medical condition.

## Data Availability

The original contributions presented in the study are included in the article/supplementary material, further inquiries can be directed to the corresponding author/s.
